# Positive association of triglyceride-glucose index with new-onset hypertension among adults: a national cohort study in China

**DOI:** 10.1186/s12933-023-01795-7

**Published:** 2023-03-16

**Authors:** Qi Gao, Yuxin Lin, Ruqi Xu, Fan Luo, Ruixuan Chen, Pingping Li, Yuping Zhang, Jiao Liu, Zhenan Deng, Yanqin Li, Licong Su, Sheng Nie

**Affiliations:** 1 Division of Nephrology, National Clinical Research Center for Kidney Disease, State Key Laboratory of Organ Failure Research, Nanfang Hospital, Southern Medical University, 1838 N Guangzhou Ave, Guangzhou, 510515 China; 2 State Key Laboratory of Respiratory Disease, National Clinical Research Center for Respiratory Disease, Guangzhou Institute of Respiratory Health, The First Affiliated Hospital of Guangzhou Medical University, Guangzhou, Guangdong China

**Keywords:** TyG index, Blood pressure, New-onset hypertension, CHNS, Prospective cohort study

## Abstract

**Background:**

Previous studies showed that the triglyceride-glucose (TyG) index was a better predictor of adverse cardiovascular events than triglycerides or fasting blood glucose alone. However, few studies have focused on new-onset hypertension. We aimed to explore the association of TyG index with new-onset hypertension in Chinese adults.

**Methods:**

A total of 4,600 participants who underwent at least 2 rounds of visits from 2009 to 2015 in the China Health and Nutrition Survey were enrolled in this study. Our outcome of interest was new-onset hypertension. Multivariate Cox hazard regression models and restricted cubic spline were performed to explore the relationship between TyG index and new-onset hypertension.

**Results:**

The mean (standard deviation, SD) age of the study population was 48.1 (13.6) years, and 2058 (44.7%) of the participants were men. The mean (SD) TyG index level was 8.6 (0.7). A total of 1,211 (26.3%) participants developed new-onset hypertension during a median (interquartile range) follow-up duration of 6.0 (2.0–6.1) years. The incidences of new-onset hypertension were 18.1%, 25.3%, 28.5%, and 33.4% by quartiles of TyG index [from quartile 1 (Q1) to Q4], respectively. The Cox model showed that high levels of TyG index were significantly associated with increased risk of new-onset hypertension (adjusted hazard ratio [aHR]: 1.29, 95% confidence interval [CI] 1.07–1.55, Q2; aHR, 1.24, 95% CI 1.03–1.49, Q3; aHR, 1.50, 95% CI 1.22–1.84, Q4) compared with Q1. Consistently, as a continuous variable, for every 1.0 increase in TyG index, there was a 17% increase in the risk of new-onset hypertension (aHR, 1.17; 95% CI 1.04–1.31). The associations were consistent in various subgroups and sensitivity analysis. The dose–response curve indicated a positive, linear association between TyG index and the risk of new-onset hypertension.

**Conclusions:**

High TyG index was significantly associated with an increased risk of new-onset hypertension among Chinese adults. Our findings suggest that maintaining a relatively low level of TyG index might be effective in the primary prevention of hypertension.

**Supplementary Information:**

The online version contains supplementary material available at 10.1186/s12933-023-01795-7.

## Introduction

Hypertension is the leading cause of cardiovascular events and all-cause mortality worldwide, which has become an emerging challenge for global public health [1]. In China, with an aging population and changing lifestyles, the prevalence of hypertension is also increasing year by year, with approximately one-third of the adult population, or more than 300 million people, suffering from hypertension between 2014 and 2015 [2, 3]. Therefore, early identifying the high-risk individuals and developing effective primary prevention strategies are very urgent to reverse the rapidly rising trend of hypertension.

Disorders of lipoprotein metabolism, in particular elevated plasma triglycerides (TG), and elevated fasting blood glucose (FBG), are all established risk factors for cardiovascular disease, especially in hypertension [4, 5]. This could be explained by insulin resistance (IR) via at least three mechanisms: inflammatory endothelial dysfunction [6], ectopic synthesis of angiotensinogen [7], and hyperinsulinaemia overstimulating the renin-angiotensin–aldosterone system [8]. Recently, the triglyceride-glucose (TyG) index, which was calculated by using TG and FBG [9], has been proposed as a surrogate of IR, and correlated with various indices of IR [10, 11]. Previous research, however, has primarily focused on the association between TyG index and the incidence of prediabetes [12], diabetes [13], cardiovascular events [14, 15], and all-cause mortality [16, 17]. Very limited studies were conducted to explore the association of TyG index with new-onset hypertension [18–28]. More importantly, these studies found inconsistence in their findings, including positive [18–24, 27] and non-significant [25, 26] associations. In addition, these studies were either limited to cross-sectional study designs, or single-center studies, or did not assess TyG index as a continuous variable. Only three prospective studies reported a positive association of TyG index and hypertension [18, 19, 28]. However, these two studies were both single-center studies and lacked regional representation. Although another study was a national cohort, it was limited to middle-aged and older adults aged ≥ 45 years. To validate these findings, a national, large sample, and prospective study cohort are required.

Based on data from the China Health and Nutrition Survey (CHNS) [29], we aimed to evaluate the potential associations between TyG index and new-onset hypertension in general Chinese adults to fill these important knowledge gaps.

## Methods

### Study design, population, and data source

The study population was drawn from the CHNS cohort, which has been described previously elsewhere [29]. The data, as well as study materials that support the findings of this study, are available at the CHNS website (http://www.cpc.unc.edu/projects/china). Briefly, the CHNS is an ongoing, large-scale, prospective, multistage cohort established in 1989 among the Chinese population. By 2015, the longitudinal study had enrolled 42,829 people from 388 communities in 15 provinces and autonomous cities/districts. The provinces included in the CHNS constituted 47% of China’s population [30]. To date, 10 follow-up waves (in 1989, 1991, 1993, 1997, 2000, 2004, 2006, 2009, 2011, and 2015) have been completed. At each survey round of follow-up, information on demographics, socioeconomics, diet, lifestyle habits (including smoking and alcohol consumption), and medical health was recorded by trained personnel.

**Fig. 1:**
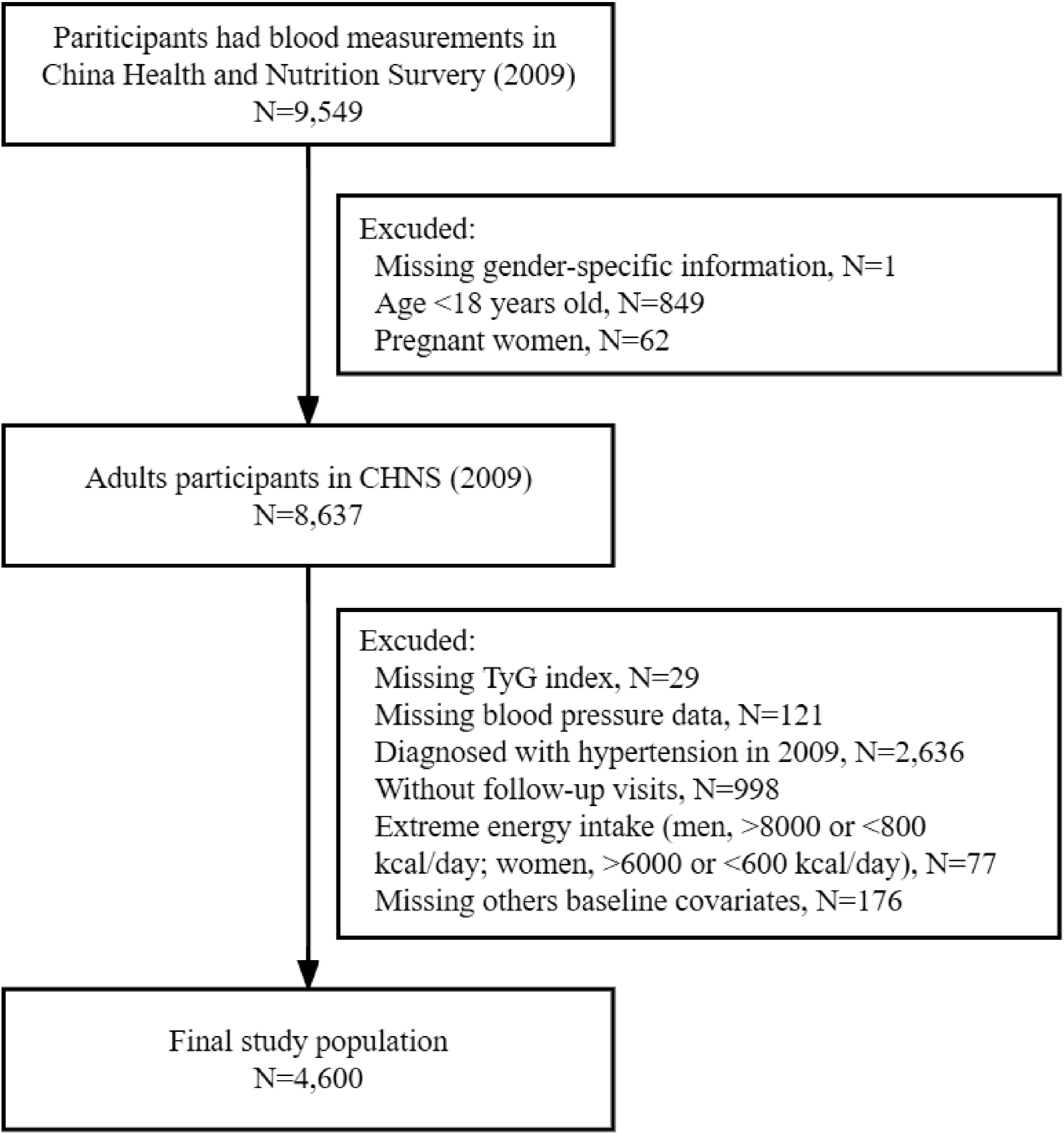
Flowchart of participants selection. *Q* quartile, *TyG* triglyceride-glucose, *CHNS* China Health and Nutrition Survey

Since blood measurements were first available in 2009, we utilized 3 rounds of CHNS data from 2009 to 2015 in this current study. Among 9,549 eligible participants in 2009, we first excluded those without gender-specific information (n = 1), younger than 18 years (n = 849), or who were pregnant (n = 62). Of the 8,637 participants, we further excluded participants without TyG index (n = 29) or blood pressure data (n = 121). In addition, participants who were diagnosed with hypertension in 2009 (n = 2,636) or who had no follow-up visits (n = 998), were also excluded. Furthermore, participants who had extreme dietary energy intake (< 800 or > 8000 kcal/d for male and < 600 or > 6000 kcal/d for female) (n = 77) or without other baseline covariates (n = 176, Additional file 4: Table S1) were also excluded. Finally, a total of 4,600 participants were included in the analysis (Fig. 1).

The study was approved by the institutional review committees of the University of North Carolina at Chapel Hill, the National Institute of Nutrition and Food Safety, and the Chinese Center for Disease Control and Prevention. Each participant provided their written informed consent. The study complies with the Declaration of Helsinki and the STROBE (Strengthening the Reporting of Observational Studies in Epidemiology) statement [31] was followed in the reporting of this study.

### Standard questionnaire and examinations

A standard questionnaire was used to assess the demographics and socioeconomics data including sex, age, urban residence (yes or no), region (region was divided into north, or south based on the Qinling Mountains-Huaihe River Line.), marital status (married, unmarried, windowed, or others), education level (illiteracy, primary school, middle school, or high school or above), occupation (farmer, worker, unemployed, or others), smoking and drinking status (yes or no). Body weight and height, waist and hip circumstance and blood pressure were measured by trained study staff. Body mass index (BMI) was calculated as weight (kg)/height (m) squared. Waist to hip ratio (WHR) was calculated as waist (m)/hip (m) circumstance. Overweight was defined as BMI > 24 kg/m^2^ in Chinese adults.

The questionnaire on physician-diagnosed hypertension and antihypertensive treatment included the following questions: “(1) Has a doctor ever told you that you suffer from high blood pressure? If yes, (2) for how long have you had it? and (3) are you currently taking anti-hypertension drugs?” In China, hypertension was defined as a clinical systolic blood pressure (SBP) of 140 mmHg or greater, and/or diastolic blood pressure (DBP) of 90 mmHg or greater without the use of antihypertensive medications according to the Chinese Guidelines for Prevention and Treatment of Hypertension (1999, 2005, 2010, and 2018 versions). Overall, all the physicians used the same criteria for the clinical diagnosis and treatment of hypertension during the follow-up period.

### Dietary nutrient intakes

Individual dietary values including energy, carbohydrate, fat, and protein intakes were assessed using 3 consecutive days (randomly allocated from Monday to Sunday and equally balanced across the 7 days of the week for each sampling unit) of 24-h dietary recalls by trained nutritionists [30]. In addition, the dietary recall has been validated as an effective approach to qualifying subjects’ daily nutritional intake. Furthermore, dietary intakes in the 2009 survey were calculated using the China Food Composition Tables (FCT) version 2004. More details about the dietary recall were described on the CHNS official website.

### Blood pressure measurements

At each follow-up survey, seated blood pressure measurements were obtained by trained research staff after the participants had rested for 5 min using a mercury manometer, following the standard method with appropriately sized cuffs. Triplicate measurements on the same arm were taken in a quiet and bright room. The means of SBP and DBP of the 3 independent measures were used in the analysis.

### Blood sample collections and measurements

Participants were asked to fast for 8–12 h prior to all blood sample collections. A total of 12 mL of blood from one participant was stored and measured in the Ministry of Health laboratory of the China-Japan Friendship Hospital. TG, serum total cholesterol (TC), high density lipoprotein cholesterol (HDL-C), and low density lipoprotein cholesterol (LDL-C) were measured by the enzymatic method. FBG was measured using glucose oxidase. Lipids and glucose were analyzed with an automatic biochemical analyzer (Hitachi 7600, Kyowa, Japan). Methods for measuring other biomarkers, including glycosylated hemoglobin (HbA1c), blood urea nitrogen (BUN), uric acid, hemoglobin, serum creatinine (SCr), high sensitivity C reactive protein (hsCRP), and fasting blood insulin (FBI), have been described elsewhere [30].

Diabetes mellitus (DM) was defined as self-reported physician-diagnosed diabetes, taking oral hypoglycemic drugs/insulin injection, FBG ≥ 7.0 mmol/l, or HbA1c ≥ 6.5% by the American Diabetes Association (ADA) [32]. Chronic kidney disease (CKD) was defined as estimated glomerular filtration rate (eGFR) < 60 ml/min/1.73m^2^ using the Chronic Kidney Disease Epidemiology Collaboration equation (CKD-EPI) [33].

### Exposure

The TyG index was calculated as follows: ln (TG (mg/dL) × FBG (mg/dL) / 2) [9].

### Outcome

The study outcome was new-onset hypertension, defined as mean SBP ≥ 140 mmHg and/or mean DBP ≥ 90 mmHg, or a physician hypertension diagnosis, or undergoing treatments for hypertension during follow-up in accordance with criteria of the WHO.

### Handling of missing variables

The distribution of missing variables was shown in Additional file 4: Table S1. To account for the small proportion of missing data, we only included participants with completed data in our primary analysis. However, in order to control the impact of missing values of some biochemical indicators, random forest imputation method was used to impute the missing variables to conducted an additional analysis.

### Statistical analysis

Shapiro–Wilk’s normality test and Bartlett test were used to detect the normal distribution and homogeneity of variance of continuous variables, respectively. Continuous variables were presented as the mean ± standard deviation (SD) for normally distributed data compared using One-Way ANOVA or a median (interquartile range [IQR]) for data that were not normally distributed compared using Kruskal–Wallis test. Categorical data were presented as a number (percentage) and compared using Pearson χ^2^ test.

The study population was divided into 4 groups based on the quartiles of baseline TyG index. The 2009 survey was considered baseline, and the follow-up period started from the baseline to the date of the first occurrence of an outcome or the latest survey round (in 2015) or lost to follow-up, which came first. The new-onset hypertension incidence rate was expressed as per 1,000 person-years. The univariate and multivariate Cox proportional hazard regression models were conducted to identify the association of TyG index with new-onset hypertension, and hazard ratios (HRs) were expressed with their 95% confidence intervals (95% CI). Model 1 adjusted for sex, age, BMI, WHR, baseline SBP and DBP, and smoking/drinking status. Model 2 adjusted for, in addition to variables included in Model 1, urban residence, region, marital status, education level, occupation, dietary intakes, presence of DM, and biochemical variables (including BUN, uric acid, eGFR, hemoglobin, hsCRP, TP, HDL-C, LDL-C, HbA1c, and FBI). The proportional hazards assumption was tested by plotting Schoenfeld residuals against time, followed by a visual inspection for uniformity. The variance inflation factor (VIF) [34] for all predictors in our models was less than 5 (Additional file 1: Figure S1), indicating the absence of significant multicollinearity. We also performed restricted cubic spline (RCS) Cox regression, with 4 knots (5 h, 35th, 65th and 95th percentiles of TyG index), to test for linearity and characterize level-response relationships between TyG index and new-onset hypertension.

### Subgroup analyses

The possible modifications of the association between TyG index and new-onset hypertension were performed in several subgroups. Participants were stratified by age (< 50 vs. ≥ 50 years), sex, BMI (< 24 vs. ≥ 24 kg/m^2^), WHR (< 0.85 [median] vs. ≥ 0.85), smoking status, drinking status, SBP (< 120 vs. 120- < 140 mmHg), DBP (< 80 vs. 80- < 90 mmHg), residence (urban vs. rural), region (north vs. south), fat (< 70 [median] vs. ≥ 70 g/d), protein (< 63 [median] vs. ≥ 63 g/d), carbohydrate (< 288 [median] vs. ≥ 288 g/d), and presence CKD (yes/no). We included an interaction term in the model for each analysis to assess effect measure modification.

### Sensitivity analysis

The robustness of the study results was further verified by various sensitivity analyses. Firstly, considering that the exact time-point of outcome occurred is difficult to capture. We fitted a Cox model using interval-censoring methods [35] to assess whether results were affected. Secondly, we excluded information on hypertension obtained through questionnaires including the presence or absence of hypertension and the use of hypertensive medication, since there might be a recall bias. Thirdly, we reanalyzed the data in participants limited with two follow-up visits to test whether the length of follow-up has an effect on the outcome. In addition, we modified the diagnostic thresholds in accordance with of American College of Cardiology (ACC) and American Heart Association (AHA) guidelines [36]. In this part, hypertension was redefined as an average SBP ≥ 130 mmHg and/or an average DBP ≥ 80 mmHg, a physician hypertension diagnosis, or taking anti-hypertension medication. Finally, the propensity score (PS) of TyG index levels (Q1 vs. Q2-Q4) was estimated using a logistic regression model whose covariates were listed in Table 1, and a baseline balanced cohort was constructed using the 1:1 Propensity Score Matching (PSM) [37] method with nearest-neighbor matching without replacement and within a caliper width of 0.001. A standardized mean difference less than 0.10 was considered a satisfactory balance between the 2 groups.

**Table 1:** Baseline characteristics of participants stratified by Quartiles of TyG index

Characteristics	Overall	Quartiles of TyG index				
		Q1 (≤ 8.1)	Q2 (> 8.1 to 8.5)	Q3 (> 8.5 to 9.0)	Q4 (> 9.0)	P value
n	4600	1150	1150	1150	1150	
TyG index	8.6 (0.7)	7.8 (0.2)	8.3 (0.1)	8.7 (0.1)	9.5 (0.5)	< 0.001
Age, years	48.1 (13.6)	45.2 (14.2)	48.1 (14.1)	49.2 (12.8)	50.0 (12.6)	< 0.001
Male (%)	2058 (44.7)	483 (42.0)	475 (41.3)	494 (43.0)	606 (52.7)	< 0.001
SBP, mmHg	116.4 (11.2)	113.8 (11.6)	115.7 (11.1)	117.0 (11.2)	119.1 (10.2)	< 0.001
DBP, mmHg	76.0 (7.5)	74.5 (7.8)	75.2 (7.6)	76.3 (7.4)	77.8 (6.9)	< 0.001
BMI, kg/m^2^	22.9 (3.2)	21.6 (2.7)	22.3 (3.0)	23.2 (3.1)	24.3 (3.2)	< 0.001
WHR	0.87 (0.08)	0.85 (0.08)	0.86 (0.08)	0.87 (0.10)	0.89 (0.07)	< 0.001
Smoking, n (%)	1286 (28.0)	292 (25.4)	313 (27.2)	305 (26.5)	376 (32.7)	< 0.001
Drinking, n (%)	1480 (32.2)	353 (30.7)	333 (29.0)	365 (31.7)	429 (37.3)	< 0.001
Urban residence, n (%)	1335 (29.0)	251 (21.8)	318 (27.7)	351 (30.5)	415 (36.1)	< 0.001
Region^*^, n (%)						0.011
North	1894 (41.2)	505 (43.9)	455 (39.6)	438 (38.1)	496 (43.1)	
South	2706 (58.8)	645 (56.1)	695 (60.4)	712 (61.9)	654 (56.9)	
Education, n (%)						0.062
Illiteracy	937 (20.4)	218 (19.0)	232 (20.2)	259 (22.5)	228 (19.8)	
Primary school	927 (20.2)	238 (20.7)	232 (20.2)	211 (18.3)	246 (21.4)	
Middle school	2169 (47.2)	554 (48.2)	548 (47.7)	557 (48.4)	510 (44.3)	
High school or above	567 (12.3)	140 (12.2)	138 (12.0)	123 (10.7)	166 (14.4)	
Occupation, n (%)						< 0.001
Farmer	1420 (30.9)	424 (36.9)	367 (31.9)	351 (30.5)	278 (24.2)	
Worker	1284 (27.9)	332 (28.9)	337 (29.3)	307 (26.7)	308 (26.8)	
Unemployed	1674 (36.4)	341 (29.7)	405 (35.2)	426 (37.0)	502 (43.7)	
Others	222 (4.8)	53 (4.6)	41 (3.6)	66 (5.7)	62 (5.4)	
Marital status						0.008
Married	4033 (87.7)	1003 (87.2)	996 (86.6)	1034 (89.9)	1000 (87.0)	
Unmarried	236 (5.1)	76 (6.6)	68 (5.9)	44 (3.8)	48 (4.2)	
Widowed	239 (5.2)	51 (4.4)	58 (5.0)	53 (4.6)	77 (6.7)	
Others	92 (2.0)	20 (1.7)	28 (2.4)	19 (1.7)	25 (2.2)	
Dietary intake, g/d						
Energy	2098.0 [1712.0, 2533.0]	2091.0 [1731.5, 2513.0]	2116.5 [1687.3, 2587.8]	2091.5 [1720.0, 2500.8]	2100.5 [1701.3, 2530.8]	0.962
Fat	70.0 [50.0, 96.0]	69.0 [50.0, 95.0]	69.0 [50.0, 94.0]	70.0 [49.0, 97.0]	70.5 [50.0, 97.0]	0.809
Carbohydrate	288.0 [229.0, 359.0]	289.0 [230.3, 359.0]	290.0 [229.0, 370.0]	287.0 [228.0, 358.8]	284.0 [228.0, 352.0]	0.385
Protein	63.0 [51.0, 78.0]	63.0 [50.0, 78.0]	63.0 [50.0, 77.0]	63.0 [51.0, 78.0]	64.0 [51.0, 80.0]	0.334
Triglycerides, mmol/L	1.18 [0.81, 1.80]	0.64 [0.53, 0.74]	0.99 [0.88, 1.11]	1.44 [1.27, 1.64]	2.59 [2.12, 3.48]	< 0.001
TC, mmol/L	4.69 [4.10, 5.37]	4.24 [3.77, 4.78]	4.60 [4.04, 5.21]	4.88 [4.30, 5.45]	5.19 [4.58, 5.93]	< 0.001
LDL-C, mmol/L	2.85 [2.31, 3.45]	2.58 [2.13, 3.02]	2.87 [2.35, 3.40]	3.11 [2.56, 3.67]	2.96 [2.25, 3.68]	< 0.001
HDL-C, mmol/L	1.40 [1.18, 1.64]	1.55 [1.36, 1.80]	1.46 [1.27, 1.71]	1.36 [1.19, 1.59]	1.19 [1.01, 1.41]	< 0.001
hsCRP, mg/dL	1.00 [0.00, 2.00]	1.00 [0.00, 2.00]	1.00 [0.00, 2.00]	1.00 [0.00, 2.00]	1.00 [1.00, 3.00]	< 0.001
Hemoglobin, g/L	140.4 (20.5)	136.5 (21.0)	139.4 (20.2)	140.7 (20.4)	144.9 (19.3)	< 0.001
HbA1c, %	5.53 (0.80)	5.35 (0.50)	5.41 (0.53)	5.51 (0.62)	5.86 (1.23)	< 0.001
FBG, mmol/L	5.28 (1.31)	4.74 (0.56)	5.01 (0.68)	5.26 (0.79)	6.10 (2.11)	< 0.001
FBI, mmol/L	9.9 [7.1, 14.2]	8.0 [5.7, 10.9]	9.2 [7.0, 13.0]	10.5 [7.6, 14.6]	13.0 [9.0, 19.7]	< 0.001
Total protein, g/L	77.08 (5.12)	76.44 (4.81)	76.89 (4.99)	77.83 (5.11)	77.16 (5.44)	< 0.001
BUN, mmol/L	5.38 (1.47)	5.45 (1.59)	5.33 (1.50)	5.22 (1.38)	5.53 (1.40)	< 0.001
Uric acid, μmol/L	284.0 [231.0, 346.3]	252.0 [206.3, 307.0]	266.5 [223.0, 320.0]	284.0 [236.0, 343.0]	341.0 [282.0, 417.0]	< 0.001
eGFR, ml/min/1.73m^2^	81.1 (15.5)	84.6 (15.3)	80.8 (15.2)	79.3 (15.2)	80.0 (15.6)	< 0.001
CKD, n (%)	350 (7.6)	52 (4.5)	94 (8.2)	101 (8.8)	103 (9.0)	< 0.001
Diabetes mellitus, n (%)	350 (7.6)	15 (1.3)	33 (2.9)	69 (6.0)	233 (20.3)	< 0.001

*Q* quartile, *TyG* triglyceride-glucose, *SMD* standard mean difference, *SBP* systolic blood pressure, *DBP* diastolic blood pressure, *BMI* body mass index, *WHR* waist hip ratio, *TC* total cholesterol, *LDL-C* low density lipoprotein cholesterol, *HDL-C* high density lipoprotein cholesterol, *hsCRP* high sensitivity C reactive protein, *HbA1c* glycosylated hemoglobin A1c, *FBG* fasting blood glucose, *FBI* fasting blood insulin, *BUN* blood urea nitrogen, *eGFR* estimated glomerular filtration rate, *CKD* chronic kidney disease

^*^Region was divided into north (Heilongjiang, Liaoning, Shandong, and Henan), and south (Jiangsu, Hubei, Hunan, Guizhou, and Guangxi) based on the Qinling Mountains-Huaihe River Line

### Additional analyses

We conducted an E-value analysis [38] to assess the extent of unmeasured confounding that would be required to negate the observed results. In addition, considering the impact of DM and CKD on hypertension incidence, we further excluded 651 individuals with DM and/or CKD.

All the above statistical analyses were performed using the R software (version 4.1.2; http://www.r-project.org/). A two-sided P < 0.05 was considered to be statistically significant in all analyses.

## Results

### Study population and baseline characteristics

The flowchart of the study population selection was shown in Fig. 1. The characteristics of these included and excluded participants were summarized in Additional file 4: Table S2. The distribution of BMI, drinking, residence, and TyG index was similar between two groups. Of the 4,600 participants selected for analysis (of whom 2,058 were male [44.7%]), the mean (SD) age was 48.1 (13.6) years, and the mean (SD) baseline SBP and DBP were 116.4 (11.2) and 76.0 (7.5) mmHg, respectively. Participants who were excluded from the dataset (n = 4,949) tended to be younger, have a high proportion of illiteracy, and unemployed, and were more likely to have CKD or DM.

The demographic and baseline characteristics of the included participants stratified by the quintiles of TyG index were summarized in Table 1. The mean (SD) TyG index was 8.6 (0.7). In general, compared to the lowest quantile group, those in the high quantile were older, more likely to be male, and less likely to live in the north of China and urban residence; they had higher values of SBP, DBP, BMI, WHR, hemoglobin, total protein, triglycerides, HDL-C, LDL-C, FBG, and HbA1c, and a higher prevalence of CKD and DM (all P < 0.05). In addition, there were no significant differences in dietary intakes in these groups (all P > 0.05). The distribution of TyG index in the study population was shown in Additional file 2: Figure S2. The baseline characteristics of participants stratified by outcome were compared in Additional file 4: Table S3.

### Association between TyG index and New-Onset hypertension

**Fig. 2:**
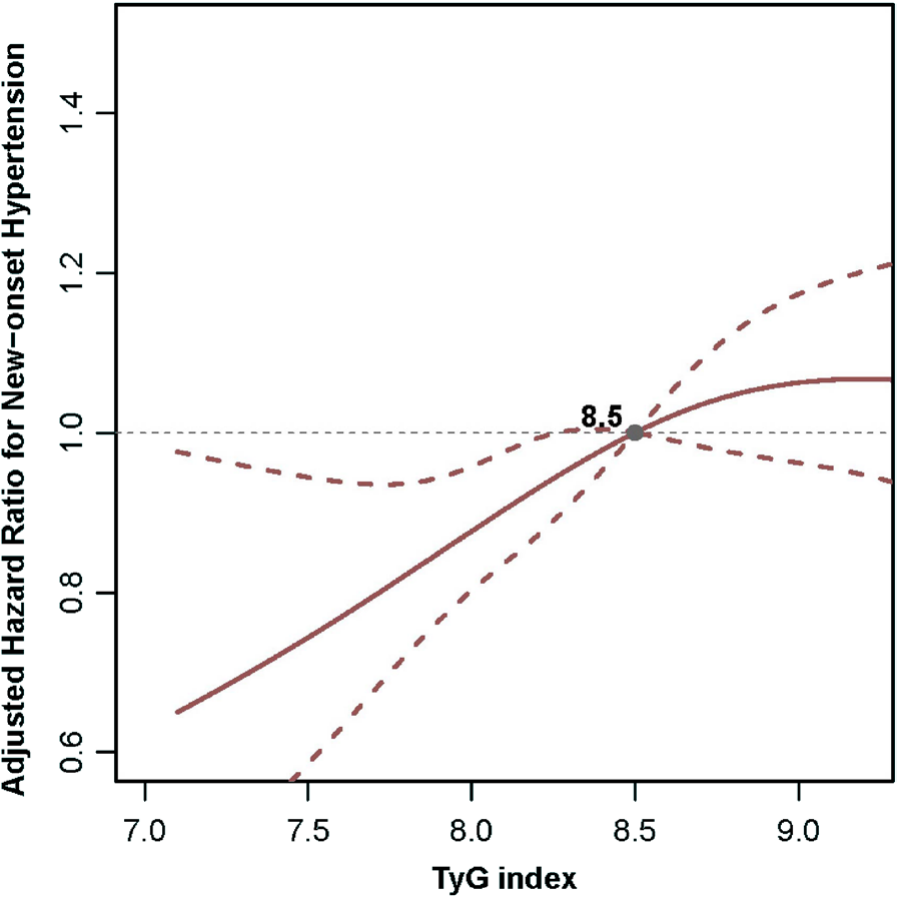
Levels of TyG index and the Risk of New-Onset Hypertension. *TyG* triglyceride-glucose, *Q* quartile, *HR* hazard ratio, *CI* confidence interval, *Ref* reference, *BMI* body mass index, *WHR* waist hip ratio, *SBP* systolic blood pressure, *DBP* diastolic blood pressure, *eGFR* estimated glomerular filtration rate, *BUN* blood urea nitrogen, *hsCRP* high sensitivity C reactive protein, *LDL-C* low density lipoprotein cholesterol, *TC* total cholesterol, *HbA1c* glycosylated hemoglobin A1c, *FBI* fasting blood insulin. The model was adjusted for sex, age, BMI, WHR, SBP, DBP, smoking, drinking, region, urban resistance, marital status, education, occupation, dietary intake of fat, protein and carbohydrate, diabetes mellitus, eGFR, BUN, uric acid, hsCRP, hemoglobin, total protein, LDL-C, TC, HbA1c, and FBI

A total of 1,211 (26.3%) participants developed new-onset hypertension during a median (IQR) follow-up duration of 6.0 (2.0–6.1) years. The incidences of new-onset hypertension were 18.1%, 25.3%, 28.5%, and 33.4% by quartiles of TyG index [from quartile 1 (Q1) to Q4], respectively. After adjusting for confounders, the Cox model showed that high levels of TyG index were significantly associated with an increased risk of new-onset hypertension (adjusted hazard ratio [aHR]: 1.29, 95% confidence interval [CI] 1.07–1.55, Q2; aHR, 1.24, 95% CI 1.03–1.49, Q3; aHR, 1.50, 95% CI 1.22–1.84, Q4) compared with Q1 (Table 2). We also found that this risk increased progressively as the TyG index increased (P for trend < 0.001). Similar trends were observed after combining the Q2–Q4 groups. Consistently in the above analysis, as a continuous variable, for per 1.0 increase in TyG index, there was a 17% increase in the risk of new-onset hypertension (aHR, 1.17; 95% CI 1.04–1.31). The dose–response curve indicated a positive, linear association between TyG index and the risk of new-onset hypertension (Fig. 2).

**Table 2:** The association of TyG index with new-onset hypertension

TyG index	Total N	No. of events (incident rate^a^)	Crude model		Model 1		Model 2	
			HR (95% CI)	P value	HR (95% CI)	P value	HR (95% CI)	P value
Quartiles								
Q1 (≤ 8.1)	1150	208 (40.4)	Ref.		Ref.		Ref.	
Q2 (> 8.1 to 8.5)	1150	291 (56.3)	1.45 (1.22–1.74)	< 0.001	1.27 (1.06–1.52)	0.010	1.29 (1.07–1.55)	0.006
Q3 (> 8.5 to 9.0)	1150	328 (62.5)	1.62 (1.37–1.93)	< 0.001	1.23 (1.03–1.47)	0.023	1.24 (1.03–1.49)	0.024
Q4 (> 9.0)	1150	384 (79.8)	2.21 (1.86–2.62)	< 0.001	1.47 (1.23–1.76)	< 0.001	1.50 (1.22–1.84)	< 0.001
P for trend				< 0.001		< 0.001		< 0.001
Categories								
Q1 (≤ 8.1)	1150	208 (40.4)	Ref.		Ref.		Ref.	
Q2-Q4 (> 8.1)	3450	1003 (65.9)	1.74 (1.50–2.02)	< 0.001	1.31 (1.12–1.53)	< 0.001	1.30 (1.11–1.53)	0.001
Continuous								
Per 1.0 increase	4600	1211 (59.4)	1.39 (1.29–1.50)	< 0.001	1.14 (1.05–1.24)	0.002	1.17 (1.04–1.31)	0.007

Model1: adjusted for sex, age, BMI, WHR, SBP, DBP, smoking, and drinking

Model2 (Full model): Model1 + further adjusted for region, urban resistance, marital status, education, occupation, dietary intake of fat, protein and carbohydrate, diabetes mellitus, eGFR, BUN, uric acid, hsCRP, hemoglobin, total protein, LDL-C, TC, HbA1c, and FBI

*TyG* triglyceride-glucose, *Q* quartile, *HR* hazard ratio, *CI* confidence interval, *Ref* reference, *BMI* body mass index, *WHR* waist hip ratio, *SBP* systolic blood pressure, *DBP* diastolic blood pressure, *eGFR* estimated glomerular filtration rate, *BUN* blood urea nitrogen, *hsCRP* high sensitivity C reactive protein, *LDL-C* low density lipoprotein cholesterol, *TC* total cholesterol, *HbA1c* glycosylated hemoglobin A1c, *FBI* fasting blood insulin

^a^Incident rate was presented as per 1000 person-years of follow-up

### Subgroup analyses

**Fig. 3:**
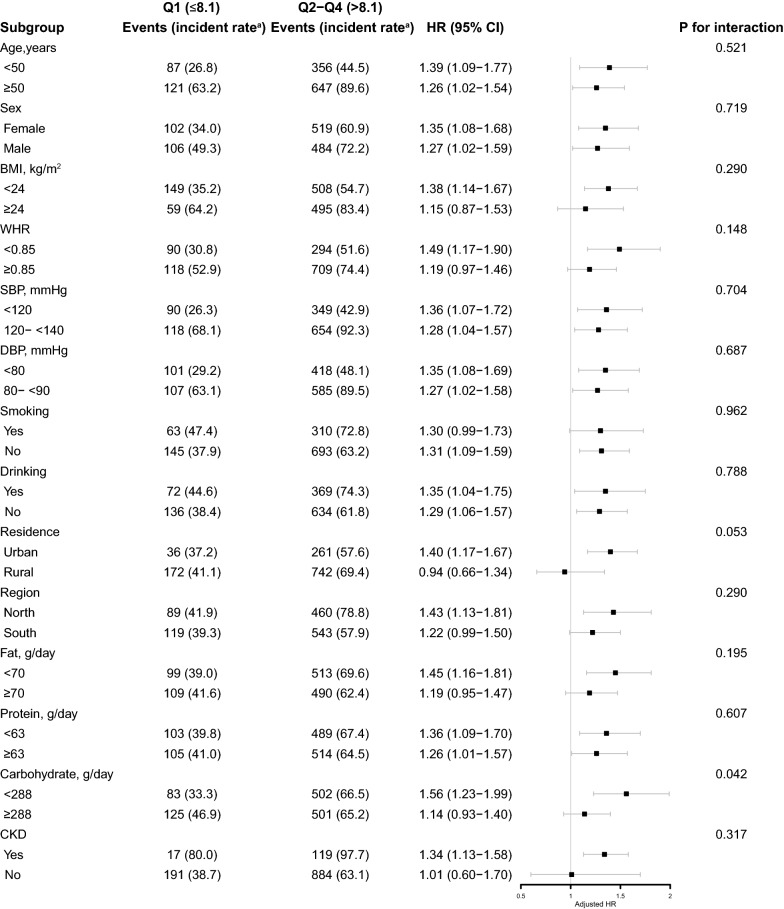
Stratified Analyses by Potential Modifiers of the Association Between TyG index and New-Onset Hypertension. *TyG* triglyceride-glucose, *Q* quartile, *HR* hazard ratio, *CI* confidence interval, *Ref* reference, *BMI* body mass index, *WHR* waist hip ratio, *SBP* systolic blood pressure, *DBP* diastolic blood pressure, *eGFR* estimated glomerular filtration rate, *BUN* blood urea nitrogen, *hsCRP* high sensitivity C reactive protein, *LDL-C* low density lipoprotein cholesterol, *TC* total cholesterol, *HbA1c* glycosylated hemoglobin A1c, *FBI* fasting blood insulin. ^a^Incident rate was presented as per 1000 person-years of follow-up. The model was adjusted for, if not stratified, sex, age, BMI, WHR, SBP, DBP, smoking, drinking, region, urban resistance, marital status, education, occupation, dietary intake of fat, protein and carbohydrate, diabetes mellitus, eGFR, BUN, uric acid, hsCRP, hemoglobin, total protein, LDL-C, TC, HbA1c, and FBI

Stratified analyses were conducted to further explore the association between TyG index (Q2-Q4 vs. Q1) and the risk of new-onset hypertension in various subgroups (Fig. 3). Notably, there was a stronger positive association in the carbohydrate intake subgroups (288 vs. ≥ 288 g/day, aHR, 1.56, 95% CI 1.23–1.99 vs. aHR, 1.14, 95% CI 0.93–1.40) (P for interaction = 0.042). None of the other variables, including age, sex, BMI, baseline SBP and DBP, smoking and drinking status, residence, regions, fat intake, protein intake, and presence of CKD or DM, significantly modified this relationship (P for interaction > 0.05).

### Sensitivity analyses

The results remained consistent when several methods were utilized to verify the robustness of the relationship between TyG index and new-onset hypertension.

The effect of TyG index on new-onset hypertension was consistent when the interval-censoring Cox model was utilized for analysis (Q2-Q4 vs. Q1, aHR, 1.30; 95% CI 1.13–1.47; per 1.0 increase, aHR, 1.14; 95% CI 1.02–1.26) (Additional file 4: Table S4). After excluding the questionnaire data defining hypertension, the results were not substantially changed (Q2-Q4 vs. Q1, aHR, 1.24; 95% CI 1.06–1.45; per 1.0 increase, aHR, 1.05; 95% CI 1.03–1.28) (Additional file 4: Table S5). In addition, when we restricted participants to only two follow-up visits, the result was consistent with the main analysis (Q2-Q4 vs. Q1, aHR, 1.32; 95% CI 1.12–1.57; per 1.0 increase, aHR, 1.15; 95% CI 1.02–1.29) (Additional file 4: Table S6). Similar trends were observed between TyG index and new-onset hypertension after redefining hypertension by the new ACC and AHA guidelines with a BP threshold of 130/80, although there was no significantly statistical difference (Q2-Q4 vs. Q1, aHR, 1.09; 95% CI 0.94–1.26; per 1.0 increase, aHR, 0.98; 95% CI 0.87–1.09) (Additional file 4: Table S7). Finally, after conducting 1:1 PSM, we obtained 993 pairs of subjects with baseline-balanced in Q1 and Q2-Q4 groups (Additional file 4: Table S8). The Cox model also showed that high levels of TyG index were significantly associated with a higher risk of new-onset hypertension (Q2-Q4 vs. Q1, aHR, 1.25; 95% CI 1.02–1.52; per 1.0 increase, aHR, 1.23; 95% CI 1.03–1.48) (Additional file 4: Table S9).

### Additional analyses

The E-values for the hazard ratio and lower confidence bound for the outcome were 1.69 and 1.36 (Additional file 3: Figure S3). After excluding 651 individuals with DM and/or CKD, or imputing the missing variables, the results were consistent with the primary analysis (Additional file 4: Tables S10, S11).

## Discussion

In this large, national, longitudinal cohort study among general Chinese adults, containing 4,600 participants with up to 6 years of follow-up, we found a positive association between TyG index and new-onset hypertension after adjusting for confounders, and this relationship was consistent across various subgroups and in sensitivity analyses. The dose–response curve indicated a positive, linear association between TyG index and the risk of new-onset hypertension. To our best knowledge, this is the first national prospective study with adults of all ages (18–94 years old) to investigate the relationship between TyG index and new-onset hypertension. The present study may provide new insights into the primary prevention of hypertension.

TyG index, as a product of FBG and triglyceride, has been confirmed to date to be closely related to the traditional risk factors of cardiovascular disease [39]. Sanchez-Inigo L et al. [40] reported, during a 10-year follow-up, that a high TyG index was significantly associated with an increased risk of future ASCVD events. Similarly, two Korean studies found TyG index to be an independent predictor of progression of coronary artery calcification [14, 41]. These evidences suggested that TyG index could have an indirect effect on cardiovascular disease.

The effects of TyG index on blood pressure have been evaluated in several previous studies, which have reported inconsistent results [18–27, 42]. Several cross-sectional studies have shown that an elevated TyG index was associated with an increased risk of developing hypertension, either in the general population [20–23], in children or adolescents [42], or in the elderly population [24]. Moreover, a prospective study in Spanish [18] reported a positive association between TyG index and hypertension in the general population during a long-term follow-up. Zheng et al. [19] also conducted another single-center, longitudinal study with 4,686 subjects followed up for 9 years, and demonstrated that TyG index could predict incident hypertension among the Chinese population. Of note, another study [28] reached a similar conclusion using a national cohort, but it was limited to middle-aged and older adults over 45 years. In addition, in a recent meta-analysis of 8 studies involving 200,044 general adult participants [43], the relative risk of hypertension was higher for the highest category of TyG index compared with the lowest. These studies were consistent with our study, which showed that high levels of TyG index were significantly associated with increased risk of new-onset hypertension and that a linear association was observed. Contrary to previous studies and our results, two cross-sectional studies [25, 26] did not find a significant association between TyG index and hypertension in obese or normal‐weight individuals. This may be attributed to the heterogeneity of the selected population and the sample size of the study. Therefore, further research on this topic is needed.

In stratified analysis, we found that most of the variables did not significantly modify the association between TyG index and new-onset hypertension, which indicates that the results of this study are applicable to the majority of the general population. However, the association between TyG index and new-onset hypertension was stronger in patients with low carbohydrate intake (< 288 g/day). Previous studies have reported that higher carbohydrate intake was related to a higher risk of hypertension [44, 45]. Therefore, individuals with higher carbohydrate intake will offset some of the risk from TyG index.

The potential mechanisms for the association between TyG index and new-onset hypertension may be explained by IR. IR has been confirmed by many studies to be significantly associated with hypertension by many mechanisms [6–8]. In recent years, many studies have concluded that TyG index is a surrogate of IR because it was correlated with various indices of IR, such as the M rates in the hyperinsulinaemic–euglycaemic clamp test [10] or the homeostasis of minimal assessment of insulin resistance (HOMA-IR) [46], and with the degree of carotid atherosclerosis [11]. More mechanistic studies are needed to further validate the relationship between TyG index, IR and hypertension.

The major strengths of this study were the 6-year national, longitudinal population-based study and the large number of subjects used to explore the relationship between TyG index and new-onset hypertension. However, certain limitations also existed in this study. First, although we adjusted for potential confounders such as demographics, dietary intake, and blood biochemical markers as much as possible, the E-values for the hazard ratio and lower confidence bound for the primary outcome were also small, which implies that little unmeasured confounding would be needed to reduce the observed association or its 95% CI to the null. However, residual confounding could not be completely eliminated. Second, limited by observational studies, we could not determine the causal relationship between TyG index and new-onset hypertension. Third, since the definition of hypertension was based on physician on-site blood pressure measurement data and questionnaires, there may be recall bias, but the findings remained consistent when we excluded questionnaire-based information on hypertension obtained in the sensitivity analysis. Forth, we could not explore the relationship between TyG index and different hypertension subtypes since information related to 24-h dynamic changes in blood pressure was not available. Fifth, considering that death was an inevitable competitive risk, we may underestimate the relationship between TyG index and new-onset hypertension. However, in our study population, only 74 (1.6%) participants died during the follow-up, which is unlikely to change the trend of results. Last, this study was limited to the Chinese general population, and more studies were needed to confirm the consistency of findings in other ethnic and national studies in the future.

## Conclusions

In conclusion, we confirmed that high levels of TyG index were associated with higher risk of new-onset hypertension and showed a linear relationship through a longitudinal national cohort. Our findings suggest that maintaining a relatively low level of TyG index will help with the primary prevention of hypertension. In clinical practice, the TyG index is easily available, and clinicians could use this indicator to risk-stratify the general population in order to provide more personalized prevention or treatment.

## Supplementary Information

**Additional file 1: Figure S1.** The variance inflation factor (VIF) values for all variables in our model.

**Additional file 2: Figure S2.** Distribution of TyG index in the study population.

**Additional file 3: Figure S3.** E-value analysis to assess the extent of unmeasured confounding that would be required to negate the observed results.

**Additional file 4: Table S1.** Distribution of missing variables. **Table S2.** Baseline characteristics of excluded and included participants. **Table S3.** Baseline characteristics of participants stratified by outcome. **Table S4.** The association of TyG index with new-onset hypertension using interval censored Cox regression model. **Table S5.** The association of TyG index with new-onset hypertension after excluding the questionnaire data defining hypertension. **Table S6.** The association of TyG index with new-onset hypertension in participants limited with two follow-up visits. **Table S7.** The association of TyG index with new-onset hypertension defined by the novel diagnostic criteria (SBP/DBP: 130/80). **Table S8.** Baseline characteristics of participants stratified by TyG index quartiles after 1:1 propensity score matching. **Table S9.** The association of TyG index with new-onset hypertension after 1:1 propensity score matching. **Table S10.** The association of TyG index with new-onset hypertension after excluding individuals with DM and/or CKD. **Table S11.** The association of TyG index with new-onset hypertension after imputing the baseline missing values.

## Data Availability

The datasets generated and/or analysed during the current study are available in the China Health and Nutrition Survey repository [http://www.cpc.unc.edu/projects/china].

## Abbreviations

TyG index: Triglyceride-glucose index
CHNS: China Health and Nutrition Survey
SD: Standard deviation
Q1: Quartile 1
Q2: Quartile 2
Q3: Quartile 3
Q4: Quartile 4
HR: Hazard ratio
aHR: Adjusted hazard ratio
TG: Triglycerides
FBG: Fasting blood glucose
IR: Insulin resistance
STROBE: Strengthening the Reporting of Observational Studies in Epidemiology
BMI: Body mass index
WHR: Waist to hip ratio
SBP: Systolic blood pressure
DBP: Diastolic blood pressure
TC: Total cholesterol
HDL-C: High density lipoprotein cholesterol
LDL-C: Low density lipoprotein cholesterol
HbA1c: Glycosylated hemoglobin
BUN: Blood urea nitrogen
SCr: Serum creatinine
hsCRP: High sensitivity C reactive protein
FBI: Fasting blood insulin
DM: Diabetes mellitus
ADA: American Diabetes Association
CKD: Chronic kidney disease
eGFR: Estimated glomerular filtration rate
CKD-EPI: Chronic Kidney Disease Epidemiology Collaboration equation
WHO: World Health Organization
VIF: Variance inflation factor
RCS: Restricted cubic spline
IQR: Interquartile range
ACC: American College of Cardiology
AHA: American Heart Association
PS: Propensity score
PSM: Propensity Score Matching
ASCVD: Atherosclerotic Cardiovascular Disease
HOMA-IR: Homeostasis of minimal assessment of insulin resistance
