# Supplementary figures and images for: Positive association of triglyceride-glucose index with new-onset hypertension among adults: a national cohort study in China
Source: Cardiovasc Diabetol. 2023 Mar 16;22:58. doi: 10.1186/s12933-023-01795-7 (PMC10022268; doi:10.1186/s12933-023-01795-7)

The VIF values for all variables in the model

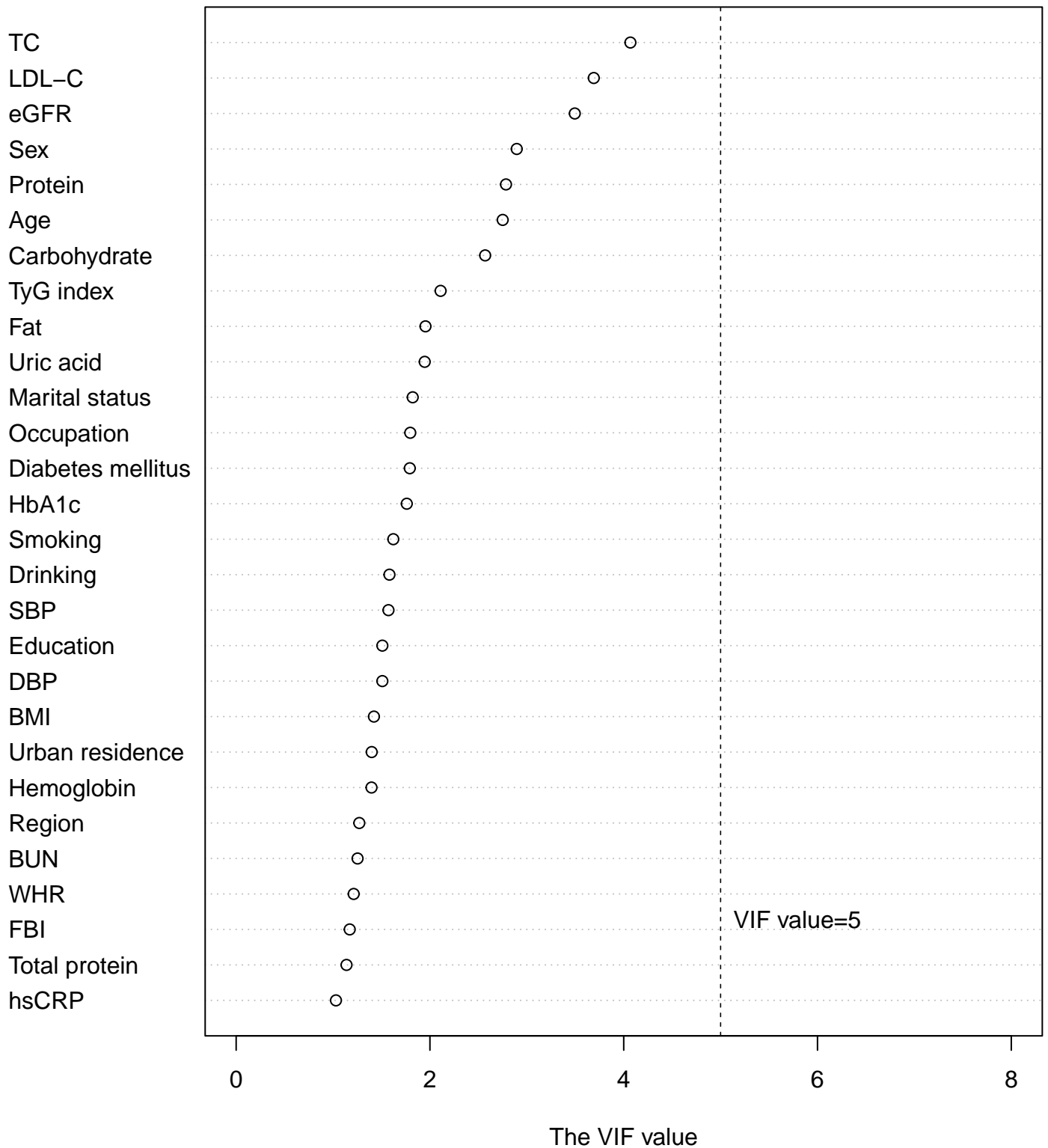

Supplement: Supplementary file 1 — Additional file 1: Figure S1. The variance inflation factor (VIF) values for all variables in our model. [file 12933_2023_1795_MOESM1_ESM.pdf]

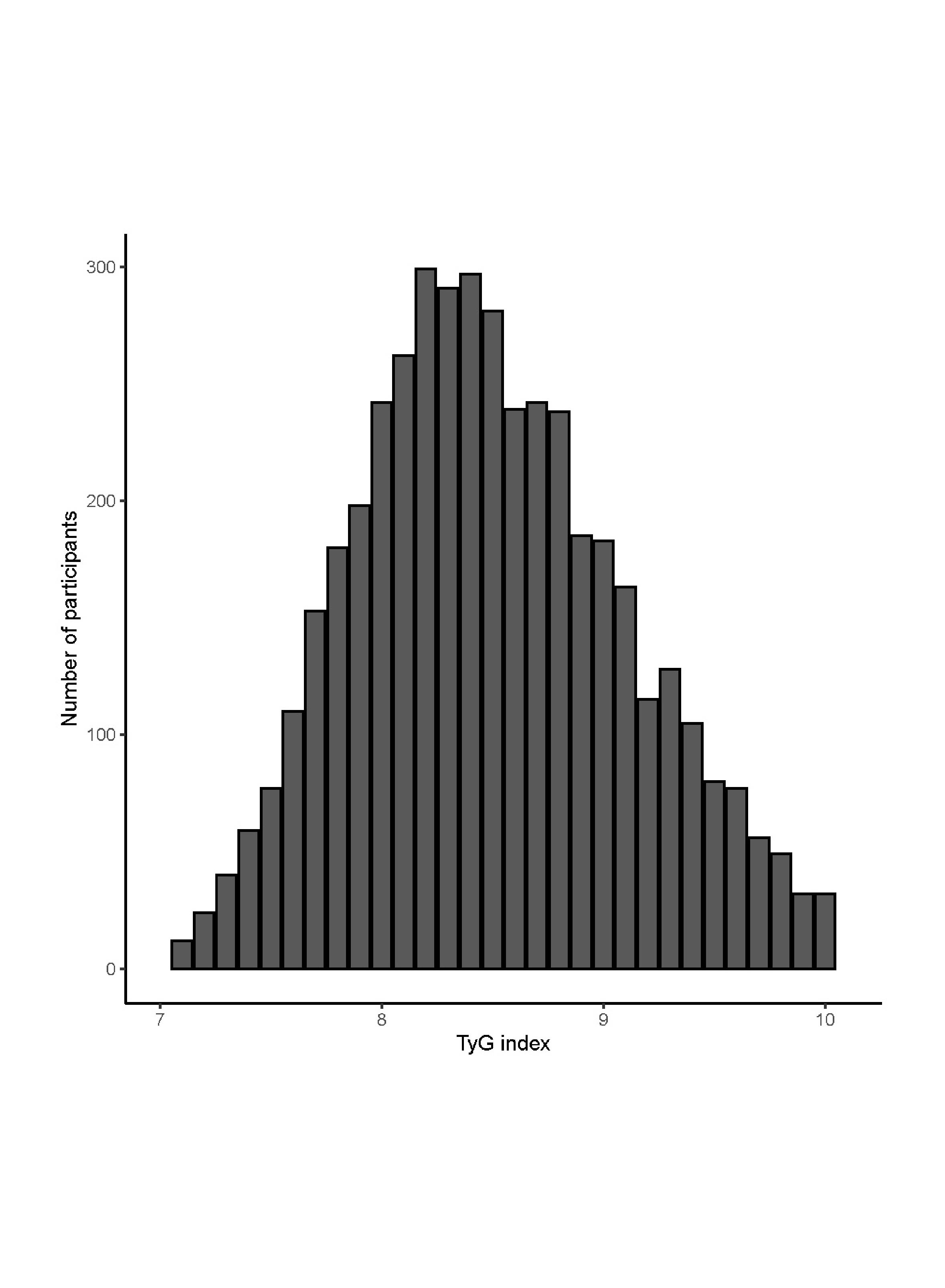

Supplement: Supplementary file 2 — Additional file 2: Figure S2. Distribution of TyG index in the study population. [file 12933_2023_1795_MOESM2_ESM.jpg]
