# Supplementary material for: Positive association of triglyceride-glucose index with new-onset hypertension among adults: a national cohort study in China
Source: Cardiovasc Diabetol. 2023 Mar 16;22:58. doi: 10.1186/s12933-023-01795-7 (PMC10022268; doi:10.1186/s12933-023-01795-7)
Supplement: Supplementary file 3 — Additional file 3: Figure S3. E-value analysis to assess the extent of unmeasured confounding that would be required to negate the observed results. [file 12933_2023_1795_MOESM3_ESM.pdf]

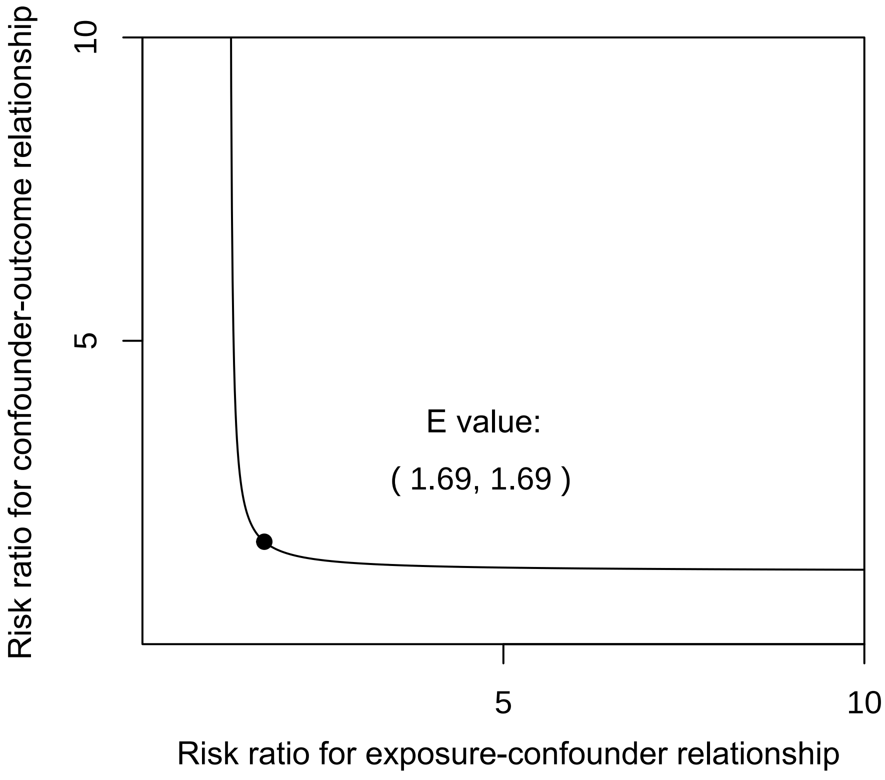
Risk ratio for confounder-outcome relationship
10
5
E value:
(1.69, 1.69)
5
10
Risk ratio for exposure-confounder relationship
