# Supplementary material for: Positive association of triglyceride-glucose index with new-onset hypertension among adults: a national cohort study in China
Source: Cardiovasc Diabetol. 2023 Mar 16;22:58. doi: 10.1186/s12933-023-01795-7 (PMC10022268; doi:10.1186/s12933-023-01795-7)
Supplement: Supplementary file 4 — Additional file 4: Table S1. Distribution of missing variables. Table S2. Baseline characteristics of excluded and included participants. Table S3. Baseline characteristics of participants stratified by outcome. Table S4. The association of TyG index with new-onset hypertension using interval censored Cox regression model. Table S5. The association of TyG index with new-onset hypertension after excluding the questionnaire data defining hypertension. Table S6. The association of TyG index with new-onset hypertension in participants limited with two follow-up visits. Table S7. The association of TyG index with new-onset hypertension defined by the novel diagnostic criteria (SBP/DBP: 130/80). Table S8. Baseline characteristics of participants stratified by TyG index quartiles after 1:1 propensity score matching. Table S9. The association of TyG index with new-onset hypertension after 1:1 propensity score matching. Table S10. The association of TyG index with new-onset hypertension after excluding individuals with DM and/or CKD. Table S11. The association of TyG index with new-onset hypertension after imputing the baseline missing values. [file 12933_2023_1795_MOESM4_ESM.docx]

**Additional file Content**

**Additional Tables**

**Additional Table 1.** Distribution of missing variables.

**Additional Table 2.** Baseline characteristics of excluded and included participants.

**Additional Table 3.** Baseline characteristics of participants stratified by outcome.

**Additional Table 4.** The association of TyG index with new-onset hypertension using interval censored Cox regression model.

**Additional Table 5.** The association of TyG index with new-onset hypertension after excluding the questionnaire data defining hypertension.

**Additional Table 6.** The association of TyG index with new-onset hypertension in participants limited with two follow-up visits.

**Additional Table 7.** The association of TyG index with new-onset hypertension defined by the novel diagnostic criteria (SBP/DBP: 130/80).

**Additional Table 8.** Baseline characteristics of participants stratified by TyG index quartiles after 1:1 propensity score matching.

**Additional Table 9.** The association of TyG index with new-onset hypertension after 1:1 propensity score matching.

**Additional Table 10.** The association of TyG index with new-onset hypertension after excluding individuals with DM and/or CKD.

**Additional Table 11.** The association of TyG index with new-onset hypertension after imputing the baseline missing values.

**Additional Figures**

**Additional Figure** **1.** The variance inflation factor (VIF) values for all variables in our model.

**Abbreviations**: Q, quartile; TyG, triglyceride-glucose; SBP, systolic blood pressure; DBP, diastolic blood pressure; BMI, body mass index; WHR, waist hip ratio; TC, total cholesterol; LDL-C, low density lipoprotein cholesterol; hsCRP, high sensitivity C reactive protein; HbA1c, glycosylated hemoglobin A1c; FBI, fasting blood insulin; BUN, blood urea nitrogen; eGFR, estimated glomerular filtration rate.

**Model**: adjusted for sex, age, BMI, WHR, SBP, DBP, smoking, drinking, region, urban resistance, marital status, education, occupation, dietary intake of fat, protein and carbohydrate, diabetes mellitus, eGFR, BUN, uric acid, hsCRP, hemoglobin, total protein, LDL-C, TC, HbA1c, and FBI.

**Additional Figure** **2.** Distribution of TyG index in the study population.

**Additional Figure** **3.** E-value analysis to assess the extent of unmeasured confounding that would be required to negate the observed results.

**Supplemental Table 1.** Distribution of missing variables.

| Characteristic | Number of participants (N=176) |
| --- | --- |
| BMI | 31 |
| WHR | 86 |
| HbA1c | 26 |
| Hemoglobin | 12 |
| FBI | 5 |
| LDL-C | 1 |
| Smoking status | 2 |
| Drinking status | 1 |
| Education | 4 |
| Occupation | 7 |
| Marital status | 1 |

**Abbreviations**: BMI, body mass index; WHR, waist hip ratio; HbA1c, hemoglobin A1c; FBI, fasting blood insulin; LDL-C, low density lipoprotein cholesterol.

**Supplemental Table 2.** Baseline characteristics of excluded and included participants.

|  | Overall | **Excluded** | **Included** | P value |
| --- | --- | --- | --- | --- |
| n | 9549 | 4949 | 4600 |  |
| TyG index | 8.6 (0.7) | 8.6 (0.8) | 8.6 (0.7) | <0.001 |
| Age, years old | 46.8 (18.2) | 45.6 (21.6) | 48.1 (13.6) | <0.001 |
| Male (%) | 4539 (47.5) | 2481 (50.1) | 2058 (44.7) | <0.001 |
| SBP, mmHg | 122.7 (19.9) | 128.9 (24.1) | 116.4 (11.2) | <0.001 |
| DBP, mmHg | 79.4 (11.9) | 82.7 (14.1) | 76.0 (7.5) | <0.001 |
| BMI, kg/m² | 22.9 (3.8) | 22.9 (4.4) | 22.9 (3.2) | 0.581 |
| WHR | 0.87 (0.08) | 0.87 (0.08) | 0.87 (0.08) | <0.001 |
| Smoking, n (%) | 2395 (26.4) | 1109 (24.7) | 1286 (28.0) | 0.001 |
| Drinking, n (%) | 2858 (31.5) | 1378 (30.7) | 1480 (32.2) | 0.147 |
| Urban residence, n (%) | 2859 (29.9) | 1524 (30.8) | 1335 (29.0) | 0.061 |
| Region^*^, n (%) |  |  |  | 0.228 |
| North | 3993 (41.8) | 2099 (42.4) | 1894 (41.2) |  |
| South | 5556 (58.2) | 2850 (57.6) | 2706 (58.8) |  |
| Education, n (%) |  |  |  | <0.001 |
| Illiteracy | 2320 (24.5) | 1383 (28.4) | 937 (20.4) |  |
| Primary school | 1962 (20.7) | 1035 (21.2) | 927 (20.2) |  |
| Middle school | 4142 (43.7) | 1973 (40.5) | 2169 (47.2) |  |
| High school or above | 1053 (11.1) | 486 (10.0) | 567 (12.3) |  |
| Occupation, n (%) |  |  |  | <0.001 |
| Farmer | 2387 (27.4) | 967 (23.5) | 1420 (30.9) |  |
| Worker | 2237 (25.7) | 953 (23.2) | 1284 (27.9) |  |
| Unemployed | 3681 (42.3) | 2007 (48.9) | 1674 (36.4) |  |
| Others | 403 (4.6) | 181 (4.4) | 222 (4.8) |  |
| Marital status |  |  |  | <0.001 |
| Married | 7303 (84.2) | 3270 (80.3) | 4033 (87.7) |  |
| Unmarried | 509 (5.9) | 273 (6.7) | 236 (5.1) |  |
| Widowed | 676 (7.8) | 437 (10.7) | 239 (5.2) |  |
| Others | 184 (2.1) | 92 (2.3) | 92 (2.0) |  |
| Dietary intake, g/d |  |  |  |  |
| Energy | 2037.0 [1639.0, 2482.0] | 1978.0 [1581.5, 2419.5] | 2098.0 [1712.0, 2533.0] | <0.001 |
| Fat | 68.0 [48.0, 93.0] | 66.00 [46.0, 90.0] | 70.00 [50.0, 96.0] | <0.001 |
| Carbohydrate | 277.0 [218.0, 349.0] | 266.0 [210.0, 339.0] | 288.0 [229.0, 359.0] | <0.001 |
| Protein | 62.0 [49.0, 78.0] | 60.00 [47.0, 77.0] | 63.0 [51.0, 78.0] | <0.001 |
| Triglycerides, mmol/L | 1.22 [0.82, 1.89] | 1.25 [0.83, 1.97] | 1.18 [0.81, 1.80] | 0.001 |
| TC, mmol/L | 4.68 [4.06, 5.39] | 4.67 [4.01, 5.40] | 4.69 [4.10, 5.37] | 0.042 |
| LDL-C, mmol/L | 2.83 [2.25, 3.46] | 2.79 [2.19, 3.46] | 2.85 [2.31, 3.45] | 0.003 |
| HDL-C, mmol/L | 1.38 [1.16, 1.63] | 1.36 [1.15, 1.62] | 1.40 [1.18, 1.64] | <0.001 |
| hsCRP, mg/dL | 1.00 [0.00, 2.00] | 1.00 [0.00, 3.00] | 1.00 [0.00, 2.00] | <0.001 |
| Hemoglobin, g/L | 140.9 (20.4) | 141.4 (20.3) | 140.4 (20.5) | 0.006 |
| HbA1c, % | 5.60 (0.90) | 5.66 (0.97) | 5.53 (0.80) | <0.001 |
| FBG, mmol/L | 5.36 (1.44) | 5.43 (1.54) | 5.28 (1.31) | <0.001 |
| FBI, mmol/L | 10.5 [7.4, 15.3] | 11.1 [7.8, 16.6] | 9.9 [7.1, 14.2] | <0.001 |
| Total protein, g/L | 77.1 (5.2) | 77.2 (5.2) | 77.1 (5.1) | 0.291 |
| BUN, mmol/L | 5.38 (1.60) | 5.37 (1.71) | 5.38 (1.47) | 0.852 |
| Uric acid, μmol/L | 296.0 [238.0, 362.0] | 306.0 [246.0, 373.0] | 284.0 [231.0, 346.3] | <0.001 |
| eGFR, ml/min/1.73m² | 83.7 (21.7) | 86.0 (26.1) | 81.1 (15.5) | <0.001 |
| CKD, n (%) | 962 (10.1) | 612 (12.5) | 350 (7.6) | <0.001 |
| Diabetes mellitus, n (%) | 949 (9.9) | 599 (12.1) | 350 (7.6) | <0.001 |

^*^Region was divided into north (Heilongjiang, Liaoning, Beijing, Shandong, and Henan), and south (Jiangsu, Shanghai, Hubei, Hunan, Chongqing, Guizhou, and Guangxi) based on the Qinling Mountains-Huaihe River Line.

**Abbreviations**: Q, quartile; TyG, triglyceride-glucose; SMD, standard mean difference; SBP, systolic blood pressure; DBP, diastolic blood pressure; BMI, body mass index; WHR, waist hip ratio; TC, total cholesterol; LDL-C, low density lipoprotein cholesterol; HDL-C, high density lipoprotein cholesterol; hsCRP, high sensitivity C reactive protein; HbA1c, glycosylated hemoglobin A1c; FBG, fasting blood glucose; FBI, fasting blood insulin; BUN, blood urea nitrogen; eGFR, estimated glomerular filtration rate; CKD, chronic kidney disease.

**Supplemental Table 3.** Baseline characteristics of participants stratified by outcome.

| Characteristics |  | **New-onset hypertension** | | |
| --- | --- | --- | --- | --- |
|  | Overall | **No** | **Yes** | P value |
| n | 4600 | 3389 | 1211 |  |
| TyG index | 8.6 (0.7) | 8.5 (0.7) | 8.7 (0.7) | <0.001 |
| Age, years old | 48.1 (13.6) | 46.7 (13.4) | 53.6 (12.5) | <0.001 |
| Male (%) | 2058 (44.7) | 1468 (43.3) | 590 (48.7) | 0.001 |
| SBP, mmHg | 116.4 (11.2) | 114.6 (11.0) | 121.4 (10.2) | <0.001 |
| DBP, mmHg | 76.0 (7.5) | 75.0 (7.6) | 78.7 (6.7) | <0.001 |
| BMI, kg/m² | 22.9 (3.2) | 22.6 (3.1) | 23.7 (3.3) | <0.001 |
| WHR | 0.87 (0.08) | 0.86 (0.09) | 0.88 (0.07) | <0.001 |
| Smoking, n (%) | 1286 (28.0) | 913 (26.9) | 373 (30.8) | 0.011 |
| Drinking, n (%) | 1480 (32.2) | 1039 (30.7) | 441 (36.4) | <0.001 |
| Urban residence, n (%) | 1335 (29.0) | 1038 (30.6) | 297 (24.5) | <0.001 |
| Region^*^, n (%) |  |  |  | 0.001 |
| North | 1894 (41.2) | 1345 (39.7) | 549 (45.3) |  |
| South | 2706 (58.8) | 2044 (60.3) | 662 (54.7) |  |
| Education, n (%) |  |  |  | <0.001 |
| Illiteracy | 937 (20.4) | 583 (17.2) | 354 (29.2) |  |
| Primary school | 927 (20.2) | 668 (19.7) | 259 (21.4) |  |
| Middle school | 2169 (47.2) | 1675 (49.4) | 494 (40.8) |  |
| High school or above | 567 (12.3) | 463 (13.7) | 104 (8.6) |  |
| Occupation, n (%) |  |  |  | <0.001 |
| Farmer | 1420 (30.9) | 993 (29.3) | 427 (35.3) |  |
| Worker | 1284 (27.9) | 1050 (31.0) | 234 (19.3) |  |
| Unemployed | 1674 (36.4) | 1165 (34.4) | 509 (42.0) |  |
| Others | 222 (4.8) | 181 (5.3) | 41 (3.4) |  |
| Marital status |  |  |  | <0.001 |
| Married | 4033 (87.7) | 2954 (87.2) | 1079 (89.1) |  |
| Unmarried | 236 (5.1) | 211 (6.2) | 25 (2.1) |  |
| Widowed | 239 (5.2) | 150 (4.4) | 89 (7.3) |  |
| Others | 92 (2.0) | 74 (2.2) | 18 (1.5) |  |
| Dietary intake, g/d |  |  |  |  |
| Energy | 2098.0 [1712.0, 2533.0] | 2093.0 [1720.0, 2515.0] | 2117.0 [1695.5, 2583.0] | 0.422 |
| Fat | 70.0 [50.0, 96.0] | 70.0 [50.0, 96.0] | 69.0 [49.0, 94.0] | 0.368 |
| Carbohydrate | 288.0 [229.0, 359.0] | 287.0 [229.0, 356.0] | 292.0 [228.5, 367.0] | 0.113 |
| Protein | 63.0 [51.0, 78.0] | 63.0 [51.0, 78.0] | 63.0 [51.0, 79.0] | 0.756 |
| Triglycerides, mmol/L | 1.18 [0.81, 1.80] | 1.14 [0.78, 1.73] | 1.33 [0.91, 2.01] | <0.001 |
| TC, mmol/L | 4.69 [4.10, 5.37] | 4.64 [4.06, 5.32] | 4.89 [4.26, 5.54] | <0.001 |
| LDL-C, mmol/L | 2.85 [2.31, 3.45] | 2.81 [2.27, 3.41] | 2.99 [2.41, 3.58] | <0.001 |
| HDL-C, mmol/L | 1.40 [1.18, 1.64] | 1.40 [1.19, 1.64] | 1.39 [1.16, 1.64] | 0.153 |
| hsCRP, mg/dL | 1.00 [0.00, 2.00] | 1.00 [0.00, 2.00] | 1.00 [1.00, 2.00] | <0.001 |
| Hemoglobin, g/L | 140.4 (20.5) | 139.6 (20.3) | 142.6 (20.6) | <0.001 |
| HbA1c, % | 5.53 (0.80) | 5.48 (0.72) | 5.69 (0.96) | <0.001 |
| FBG, mmol/L | 5.28 (1.31) | 5.20 (1.23) | 5.49 (1.50) | <0.001 |
| FBI, mmol/L | 9.9 [7.1, 14.2] | 9.8 [7.1, 14.0] | 10.4 [7.2, 14.8] | 0.020 |
| Total protein, g/L | 77.1 (5.1) | 77.0 (5.0) | 77.2 (5.3) | 0.403 |
| BUN, mmol/L | 5.38 (1.47) | 5.31 (1.46) | 5.59 (1.50) | <0.001 |
| Uric acid, μmol/L | 284.0 [231.0, 346.3] | 280.0 [227.0, 342.0] | 297.0 [240.5, 361.0] | <0.001 |
| eGFR, ml/min/1.73m² | 81.1 (15.5) | 82.5 (15.3) | 77.4 (15.3) | <0.001 |
| CKD, n (%) | 350 (7.6) | 214 (6.3) | 136 (11.2) | <0.001 |
| Diabetes mellitus, n (%) | 350 (7.6) | 216 (6.4) | 134 (11.1) | <0.001 |

^*^Region was divided into north (Heilongjiang, Liaoning, Beijing, Shandong, and Henan), and south (Jiangsu, Shanghai, Hubei, Hunan, Chongqing, Guizhou, and Guangxi) based on the Qinling Mountains-Huaihe River Line.

**Abbreviations**: Q, quartile; TyG, triglyceride-glucose; SMD, standard mean difference; SBP, systolic blood pressure; DBP, diastolic blood pressure; BMI, body mass index; WHR, waist hip ratio; TC, total cholesterol; LDL-C, low density lipoprotein cholesterol; HDL-C, high density lipoprotein cholesterol; hsCRP, high sensitivity C reactive protein; HbA1c, glycosylated hemoglobin A1c; FBG, fasting blood glucose; FBI, fasting blood insulin; BUN, blood urea nitrogen; eGFR, estimated glomerular filtration rate; CKD, chronic kidney disease.

**Supplemental Table 4.** The association of TyG index with new-onset hypertension using interval censored Cox regression model.

| TyG index | Total  N | No. of Events  (incident rate^a^) | **Crude model** | | **Model 1** | | **Model 2** | |
| --- | --- | --- | --- | --- | --- | --- | --- | --- |
|  |  |  | HR (95% CI) | P value | HR (95% CI) | P value | HR (95% CI) | P value |
| Categories | | | | | | | | |
| Q1 (≤8.1) | 1,150 | 208 (40.4) | Ref |  | Ref |  | Ref |  |
| Q2-Q4 (>8.1) | 3,450 | 1,003 (65.9) | 1.71 (1.56-1.86) | <0.001 | 1.26 (1.11-1.42) | <0.001 | 1.30 (1.13-1.47) | <0.001 |
| Continuous |  |  |  |  |  |  |  |  |
| Per 1.0 increase | 4,600 | 1,211 (59.4) | 1.37 (1.30-1.45) | <0.001 | 1.09 (1.00-1.18) | 0.052 | 1.14 (1.02-1.26) | 0.027 |

**Abbreviations**: TyG, triglyceride-glucose; Q, quartile; HR, hazard ratio; CI, confidence interval; Ref, reference; BMI, body mass index; WHR, waist hip ratio; SBP, systolic blood pressure; DBP, diastolic blood pressure; eGFR, estimated glomerular filtration rate; BUN, blood urea nitrogen; hsCRP, high sensitivity C reactive protein; LDL-C, low density lipoprotein cholesterol; TC, total cholesterol; HbA1c, glycosylated hemoglobin A1c; FBI, fasting blood insulin.

^a^Incident rate is presented as per 1000 person-years of follow-up.

**Model1**: adjusted for sex, age, BMI, WHR, SBP, DBP, smoking, and drinking.

**Model2 (Full model)**: Model1+further adjusted for region, urban resistance, marital status, education, occupation, dietary intake of fat, protein and carbohydrate, diabetes mellitus, eGFR, BUN, uric acid, hsCRP, hemoglobin, total protein, LDL-C, TC, HbA1c, and FBI.

**Supplemental Table 5.** The association of TyG index with new-onset hypertension after excluding the questionnaire data defining hypertension.

| TyG index | Total  N | No. of Events  (incident rate^a^) | **Crude model** | | **Model1** | | **Model2** | |
| --- | --- | --- | --- | --- | --- | --- | --- | --- |
|  |  |  | HR (95% CI) | P value | HR (95% CI) | P value | HR (95% CI) | P value |
| Categories | | | | | | | | |
| Q1 (≤8.1) | 1,209 | 228 (42.0) | Ref |  | Ref |  | Ref |  |
| Q2-Q4 (>8.1) | 3,626 | 1,041 (65.5) | 1.66 (1.44-1.92) | <0.001 | 1.24 (1.07-1.44) | 0.004 | 1.24 (1.06-1.45) | 0.007 |
| Continuous |  |  |  |  |  |  |  |  |
| Per 1.0 increase | 4,835 | 1,269 (59.5) | 1.38 (1.28-1.48) | <0.001 | 1.12 (1.03-1.21) | 0.007 | 1.05 (1.03-1.28) | 0.012 |

**Abbreviations**: TyG, triglyceride-glucose; Q, quartile; HR, hazard ratio; CI, confidence interval; Ref, reference; BMI, body mass index; WHR, waist hip ratio; SBP, systolic blood pressure; DBP, diastolic blood pressure; eGFR, estimated glomerular filtration rate; BUN, blood urea nitrogen; hsCRP, high sensitivity C reactive protein; LDL-C, low density lipoprotein cholesterol; TC, total cholesterol; HbA1c, glycosylated hemoglobin A1c; FBI, fasting blood insulin.

^a^Incident rate is presented as per 1000 person-years of follow-up.

**Model1**: adjusted for sex, age, BMI, WHR, SBP, DBP, smoking, and drinking.

**Model2 (Full model)**: Model1+further adjusted for region, urban resistance, marital status, education, occupation, dietary intake of fat, protein and carbohydrate, diabetes mellitus, eGFR, BUN, uric acid, hsCRP, hemoglobin, total protein, LDL-C, TC, HbA1c, and FBI.

**Supplemental Table 6.** The association of TyG index with new-onset hypertension in participants limited with two follow-up visits.

| TyG index | Total  N | No. of Events  (incident rate^a^) | **Crude model** | | **Model1** | | **Model2** | |
| --- | --- | --- | --- | --- | --- | --- | --- | --- |
|  |  |  | HR (95% CI) | P value | HR (95% CI) | P value | HR (95% CI) | P value |
| Categories | | | | | | | | |
| Q1 (≤8.1) | 936 | 186 (39.8) | Ref |  | Ref |  | Ref |  |
| Q2-Q4 (>8.1) | 2,806 | 906 (66.3) | 1.78 (1.52-2.08) | <0.001 | 1.33 (1.13-1.56) | 0.001 | 1.32 (1.12-1.57) | 0.001 |
| Continuous |  |  |  |  |  |  |  |  |
| Per 1.0 increase | 3,742 | 1,092 (59.6) | 1.39 (1.28-1.50) | <0.001 | 1.13 (1.04-1.24) | 0.005 | 1.15 (1.02-1.29) | 0.024 |

**Abbreviations**: TyG, triglyceride-glucose; Q, quartile; HR, hazard ratio; CI, confidence interval; Ref, reference; BMI, body mass index; WHR, waist hip ratio; SBP, systolic blood pressure; DBP, diastolic blood pressure; eGFR, estimated glomerular filtration rate; BUN, blood urea nitrogen; hsCRP, high sensitivity C reactive protein; LDL-C, low density lipoprotein cholesterol; TC, total cholesterol; HbA1c, glycosylated hemoglobin A1c; FBI, fasting blood insulin.

^a^Incident rate is presented as per 1000 person-years of follow-up.

**Model1**: adjusted for sex, age, BMI, WHR, SBP, DBP, smoking, and drinking.

**Model2 (Full model)**: Model1+further adjusted for region, urban resistance, marital status, education, occupation, dietary intake of fat, protein and carbohydrate, diabetes mellitus, eGFR, BUN, uric acid, hsCRP, hemoglobin, total protein, LDL-C, TC, HbA1c, and FBI.

**Supplemental Table 7.** The association of TyG index with new-onset hypertension defined by the novel diagnostic criteria (SBP/DBP: 130/80).

| TyG index | Total  N | No. of Events  (incident rate^a^) | **Crude model** | | **Model1** | | **Model2** | |
| --- | --- | --- | --- | --- | --- | --- | --- | --- |
|  |  |  | HR (95% CI) | P value | HR (95% CI) | P value | HR (95% CI) | P value |
| Categories | | | | | | | | |
| Q1 (≤8.0) | 625 | 271 (109.1) | Ref |  | Ref |  | Ref |  |
| Q2-Q4 (>8.0) | 1,876 | 1,069 (146.1) | 1.42 (1.24-1.62) | <0.001 | 1.15 (1.01-1.32) | 0.041 | 1.09 (0.94-1.26) | 0.260 |
| Continuous |  |  |  |  |  |  |  |  |
| Per 1.0 increase | 2,501 | 1,340 (136.7) | 1.32 (1.23-1.43) | <0.001 | 1.09 (1.00-1.19) | 0.045 | 0.98 (0.87-1.09) | 0.684 |

**Abbreviations**: TyG, triglyceride-glucose; Q, quartile; HR, hazard ratio; CI, confidence interval; Ref, reference; BMI, body mass index; WHR, waist hip ratio; SBP, systolic blood pressure; DBP, diastolic blood pressure; eGFR, estimated glomerular filtration rate; BUN, blood urea nitrogen; hsCRP, high sensitivity C reactive protein; LDL-C, low density lipoprotein cholesterol; TC, total cholesterol; HbA1c, glycosylated hemoglobin A1c; FBI, fasting blood insulin.

^a^Incident rate is presented as per 1000 person-years of follow-up.

**Model1**: adjusted for sex, age, BMI, WHR, SBP, DBP, smoking, and drinking.

**Model2 (Full model)**: Model1+further adjusted for region, urban resistance, marital status, education, occupation, dietary intake of fat, protein and carbohydrate, diabetes mellitus, eGFR, BUN, uric acid, hsCRP, hemoglobin, total protein, LDL-C, TC, HbA1c, and FBI.

**Supplemental Table 8.** Baseline characteristics of participants stratified by TyG index quartiles after 1:1 propensity score matching.

| Characteristic |  | **TyG index** | | |
| --- | --- | --- | --- | --- |
|  | Overall | **Q1 (≤8.1)** | **Q2-Q4 (>8.1)** | SMD |
| n | 1986 | 993 | 993 |  |
| TyG index | 8.2 (0.5) | 7.8 (0.2) | 8.6 (0.5) | 2.280 |
| Age, years old | 46.3 (13.9) | 46.2 (14.2) | 46.4 (13.6) | 0.017 |
| Male (%) | 837 (42.1) | 423 (42.6) | 414 (41.7) | 0.018 |
| SBP, mmHg | 114.6 (11.4) | 114.5 (11.5) | 114.7 (11.2) | 0.018 |
| DBP, mmHg | 74.7 (7.8) | 74.8 (7.7) | 74.5 (8.0) | 0.039 |
| BMI, kg/m² | 21.8 (2.7) | 21.8 (2.7) | 21.8 (2.7) | 0.025 |
| WHR | 0.85 (0.08) | 0.85 (0.08) | 0.85 (0.08) | 0.011 |
| Smoking, n (%) | 513 (25.8) | 262 (26.4) | 251 (25.3) | 0.025 |
| Drinking, n (%) | 598 (30.1) | 300 (30.2) | 298 (30.0) | 0.004 |
| Urban residence, n (%) | 452 (22.8) | 227 (22.9) | 225 (22.7) | 0.005 |
| Region^*^, n (%) |  |  |  | 0.008 |
| North | 860 (43.3) | 428 (43.1) | 432 (43.5) |  |
| South | 1126 (56.7) | 565 (56.9) | 561 (56.5) |  |
| Education, n (%) |  |  |  | 0.048 |
| Illiteracy | 330 (25.7) | 159 (24.8) | 171 (26.7) |  |
| Primary school | 667 (52.0) | 335 (52.3) | 332 (51.8) |  |
| Middle school | 285 (22.2) | 147 (22.9) | 138 (21.5) |  |
| High school or above | 234 (11.8) | 116 (11.7) | 118 (11.9) |  |
| Occupation, n (%) |  |  |  | 0.035 |
| Farmer | 717 (36.1) | 357 (36.0) | 360 (36.3) |  |
| Worker | 559 (28.1) | 286 (28.8) | 273 (27.5) |  |
| Unemployed | 620 (31.2) | 304 (30.6) | 316 (31.8) |  |
| Others | 90 (4.5) | 46 (4.6) | 44 (4.4) |  |
| Marital status |  |  |  | 0.037 |
| Married | 1748 (88.0) | 871 (87.7) | 877 (88.3) |  |
| Unmarried | 116 (5.8) | 60 (6.0) | 56 (5.6) |  |
| Widowed | 88 (4.4) | 43 (4.3) | 45 (4.5) |  |
| Others | 34 (1.7) | 19 (1.9) | 15 (1.5) |  |
| Dietary intake, g/d |  |  |  |  |
| Energy | 2102.5 [1740.0, 2539.0] | 2089.0 [1728.0, 2539.0] | 2109.0 [1749.0, 2539.0] | 0.029 |
| Fat | 70.0 [51.0, 97.0] | 69.0 [50.0, 97.0] | 71.0 [52.0, 97.0] | 0.031 |
| Carbohydrate | 289.0 [229.3, 361.0] | 289.0 [229.0, 359.0] | 288.0 [230.0, 363.0] | 0.012 |
| Protein | 63.0 [51.0, 79.0] | 62.0 [50.0, 79.0] | 65.0 [51.00, 78.0] | 0.049 |
| TC, mmol/L | 4.34 [3.87, 4.88] | 4.34 [3.89, 4.86] | 4.36 [3.84, 4.92] | 0.027 |
| LDL-C, mmol/L | 2.63 [2.20, 3.11] | 2.65 [2.24, 3.11] | 2.61 [2.17, 3.11] | 0.042 |
| HDL-C, mmol/L | 1.47 [1.26, 1.71] | 1.56 [1.38, 1.82] | 1.36 [1.17, 1.60] | 0.548 |
| hsCRP, mg/dL | 1.00 [0.00, 2.00] | 1.00 [0.00, 2.00] | 1.00 [0.00, 2.00] | 0.028 |
| Hemoglobin, g/L | 137.2 (21.0) | 137.3 (20.7) | 137.2 (21.3) | 0.006 |
| HbA1c, % | 5.37 (0.51) | 5.36 (0.50) | 5.38 (0.52) | 0.025 |
| FBI, mmol/L | 8.6 [6.3, 11.7] | 8.2 [5.9, 11.1] | 9.1 [6.7, 12.4] | 0.023 |
| Total protein, g/L | 76.7 (4.9) | 76.7 (4.8) | 76.7 (5.1) | 0.001 |
| BUN, mmol/L | 5.40 (1.50) | 5.40 (1.51) | 5.40 (1.49) | <0.001 |
| Uric acid, μmol/L | 258.0 [212.0, 311.0] | 259.0 [213.0, 312.0] | 257.0 [212.0, 310.0] | 0.004 |
| eGFR, ml/min/1.73m² | 83.5 (15.1) | 83.3 (14.7) | 83.7 (15.5) | 0.026 |
| CKD, n (%) | 108 (5.4) | 49 (4.9) | 59 (5.9) | 0.044 |
| Diabetes mellitus, n (%) | 35 (1.8) | 15 (1.5) | 20 (2.0) | 0.038 |

^*^Region was divided into north (Heilongjiang, Liaoning, Beijing, Shandong, and Henan), and south (Jiangsu, Shanghai, Hubei, Hunan, Chongqing, Guizhou, and Guangxi) based on the Qinling Mountains-Huaihe River Line.

**Abbreviations**: Q, quartile; TyG, triglyceride-glucose; SMD, standard mean difference; SBP, systolic blood pressure; DBP, diastolic blood pressure; BMI, body mass index; WHR, waist hip ratio; TC, total cholesterol; LDL-C, low density lipoprotein cholesterol; HDL-C, high density lipoprotein cholesterol; hsCRP, high sensitivity C reactive protein; HbA1c, glycosylated hemoglobin A1c; FBI, fasting blood insulin; BUN, blood urea nitrogen; eGFR, estimated glomerular filtration rate; CKD, chronic kidney disease.

**Supplemental Table 9.** The association of TyG index with new-onset hypertension after 1:1 propensity score matching.

| TyG index | Total  N | No. of Events  (incident rate^a^) | **Crude model** | | **Model 1** | | **Model 2** | |
| --- | --- | --- | --- | --- | --- | --- | --- | --- |
|  |  |  | HR (95% CI) | P value | HR (95% CI) | P value | HR (95% CI) | P value |
| Categories | | | | | | | | |
| Q1 (≤8.1) | 993 | 193 (43.2) | Ref |  | Ref |  | Ref |  |
| Q2-Q4 (>8.1) | 993 | 228 (50.1) | 1.17 (0.97-1.42) | 0.11 | 1.25 (1.03-1.52) | 0.02 | 1.25 (1.02-1.52) | 0.03 |
| Continuous |  |  |  |  |  |  |  |  |
| Per 1.0 increase | 1,986 | 421 (46.7) | 1.24 (1.04-1.47) | 0.02 | 1.21 (1.02-1.44) | 0.03 | 1.23 (1.03-1.48) | 0.03 |

**Abbreviations**: TyG, triglyceride-glucose; Q, quartile; HR, hazard ratio; CI, confidence interval; Ref, reference; BMI, body mass index; WHR, waist hip ratio; SBP, systolic blood pressure; DBP, diastolic blood pressure; eGFR, estimated glomerular filtration rate; BUN, blood urea nitrogen; hsCRP, high sensitivity C reactive protein; LDL-C, low density lipoprotein cholesterol; TC, total cholesterol; HbA1c, glycosylated hemoglobin A1c; FBI, fasting blood insulin.

^a^Incident rate is presented as per 1000 person-years of follow-up.

**Model1**: adjusted for sex, age, BMI, WHR, SBP, DBP, smoking, and drinking.

**Model2 (Full model)**: Model1+further adjusted for region, urban resistance, marital status, education, occupation, dietary intake of fat, protein and carbohydrate, diabetes mellitus, eGFR, BUN, uric acid, hsCRP, hemoglobin, total protein, LDL-C, TC, HbA1c, and FBI.

**Supplementary Table 10.** The association of TyG index with new-onset hypertension after excluding individuals with DM and/or CKD.

| TyG index | Total  N | No. of Events  (incident rate^a^) | **Crude model** | | **Model 1** | | **Model 2** | |
| --- | --- | --- | --- | --- | --- | --- | --- | --- |
|  |  |  | HR (95% CI) | P value | HR (95% CI) | P value | HR (95% CI) | P value |
| Categories | | | | | | | | |
| Q1 (≤8.0) | 988 | 168 (37.7) | Ref |  | Ref |  | Ref |  |
| Q2-Q4 (>8.0) | 2961 | 794 (75.5) | 1.64 (1.39-1.93) | <0.001 | 1.31 (1.11-1.56) | 0.002 | 1.30 (1.09-1.56) | 0.004 |
| Continuous | | | | | | | | |
| Per 1.0 increase | 3949 | 962 (54.2) | 1.48 (1.35-1.62) | <0.001 | 1.22 (1.10-1.35) | <0.001 | 1.23 (1.08-1.40) | 0.002 |

**Abbreviations**: TyG, triglyceride-glucose; DM, diabetes mellitus; CKD, chronic kidney disease; Q, quartile; HR, hazard ratio; CI, confidence interval; Ref, reference; BMI, body mass index; WHR, waist hip ratio; SBP, systolic blood pressure; DBP, diastolic blood pressure; eGFR, estimated glomerular filtration rate; BUN, blood urea nitrogen; hsCRP, high sensitivity C reactive protein; LDL-C, low density lipoprotein cholesterol; TC, total cholesterol; HbA1c, glycosylated hemoglobin A1c; FBI, fasting blood insulin.

^a^Incident rate was presented as per 1000 person-years of follow-up.

**Model1**: adjusted for sex, age, BMI, WHR, SBP, DBP, smoking, and drinking.

**Model2 (Full model)**: Model1+further adjusted for region, urban resistance, marital status, education, occupation, dietary intake of fat, protein and carbohydrate, eGFR, BUN, uric acid, hsCRP, hemoglobin, total protein, LDL-C, TC, HbA1c, and FBI.

**Supplementary Table 11.** The association of TyG index with new-onset hypertension after imputing the baseline missing values.

| TyG index | Total  N | No. of Events  (incident rate^a^) | **Crude model** | | **Model 1** | | **Model 2** | |
| --- | --- | --- | --- | --- | --- | --- | --- | --- |
|  |  |  | HR (95% CI) | P value | HR (95% CI) | P value | HR (95% CI) | P value |
| Categories | | | | | | | | |
| Q1 (≤8.1) | 1194 | 220 (41.1) | Ref |  | Ref |  | Ref |  |
| Q2-Q4 (>8.1) | 3582 | 1051 (66.6) | 1.73 (1.50-2.00) | <0.001 | 1.29 (1.11-1.50) | 0.001 | 1.28 (1.09-1.50) | 0.002 |
| Continuous | | | | | | | | |
| Per 1.0 increase | 4776 | 1271 (60.1) | 1.37 (1.28-1.47) | <0.001 | 1.12 (1.03-1.21) | 0.007 | 1.13 (1.01-1.26) | 0.027 |

**Abbreviations**: TyG, triglyceride-glucose; Q, quartile; HR, hazard ratio; CI, confidence interval; Ref, reference; BMI, body mass index; WHR, waist hip ratio; SBP, systolic blood pressure; DBP, diastolic blood pressure; eGFR, estimated glomerular filtration rate; BUN, blood urea nitrogen; hsCRP, high sensitivity C reactive protein; LDL-C, low density lipoprotein cholesterol; TC, total cholesterol; HbA1c, glycosylated hemoglobin A1c; FBI, fasting blood insulin.

^a^Incident rate was presented as per 1000 person-years of follow-up.

**Model1**: adjusted for sex, age, BMI, WHR, SBP, DBP, smoking, and drinking.

**Model2 (Full model)**: Model1+further adjusted for region, urban resistance, marital status, education, occupation, dietary intake of fat, protein and carbohydrate, eGFR, diabetes mellitus, BUN, uric acid, hsCRP, hemoglobin, total protein, LDL-C, TC, HbA1c, and FBI.
